# Supplementary material for: Reasoning about mental states under uncertainty
Source: PLoS One. 2022 Nov 9;17(11):e0277356. doi: 10.1371/journal.pone.0277356 (PMC9645647; doi:10.1371/journal.pone.0277356)
Supplement: S1 Appendix — Notes. Participants read 15 vignettes, each with 3 conditions totaling to 45 vignettes depicted here; participants only viewed one condition of each vignette (i.e., 15 vignettes). The following is examples of 45 vignette scripts used in Experiment 1. For Experiments 2 and 3, the same vignettes were used with alterations in the number of filler, consistent, or inconsistent statements. (DOCX) [file pone.0277356.s001.docx]

**Examples of Vignette Scripts**

*Notes.* Participants read 15 vignettes, each with 3 conditions totaling to 45 vignettes depicted here; participants only viewed one condition of each vignette (i.e., 15 vignettes). The following is examples of 45 vignette scripts used in Experiment 1. For Experiments 2 and 3, the same vignettes were used with alterations in the number of filler, consistent, or inconsistent statements.

**Vignette #1**

**Intermediate-Uncertainty**

Introduction: This is Lewis. Lewis has a roommate named Clara. Clara plays the piano.

Filler 1. Lewis speaks four languages because growing up, his father was a linguist who encouraged Lewis to learn multiple languages.

Filler 2. Lewis and Clara’s apartment is downtown Chicago, which can get very expensive. Luckily, their landlord covers the cost of utilities.

Consistent 1. Lewis spends most of his evenings listening to Clara play the piano. After several hours of listening to her play, Lewis often goes to the kitchen.

Filler 3. He then makes himself a cup of camomile tea to help him fall asleep.

Consistent 2. One day, Lewis came across an advertisement to join the local orchestra. Lewis took a copy of the advertisement home and gave it to Clara.

Filler 4. That night, Lewis’s mother called and when they spoke, Lewis asked his mother if they would be visiting Chicago over the holidays.

Please indicate whether the following statements are “TRUE” or a “FALSE”.

Statement #1

**Lewis believe that Clara is musically talented.**

**TRUE**

FALSE

Statement #2

**Lewis *does not* think that Clara should join the local orchestra.**

TRUE

**FALSE**

Statement #3

**Lewis knows that Clara will be a successful musician.**

**TRUE**

FALSE

Statement #4

**When Lewis listens to Clara play the piano, how does he feel?**

SURPRISED

ANGRY

**HAPPY**

SAD

FEAR

DISGUSTED

Statement #5

**If Clara joins the orchestra, Lewis will *not* purchase a ticket to her first concert.**

TRUE

**FALSE**

**Low-Uncertainty**

Introduction: This is Lewis. Lewis has a roommate named Clara. Clara plays the piano.

Consistent 1 +2. Lewis spends most of his evenings listening to Clara play the piano. After several hours of listening to her play, Lewis often smiles and applauds.

Consistent 3. When Clara’s piano needed tuning, Lewis paid to have it done professionally.

Consistent 4. Lewis regularly organizes social get-togethers in their apartment and asks Clara to play the piano for their friends.

Consistent 5. He even once videotaped Clara playing the piano and uploaded the video on YouTube for the world to see.

Consistent 6. One day, Lewis came across an advertisement to join the local orchestra. Lewis took a copy of the advertisement home and gave it to Clara.

Statement #1

**Lewis believe that Clara is musically talented.**

**TRUE**

FALSE

Statement #2

**Lewis *does not* think that Clara should join the local orchestra.**

TRUE

**FALSE**

Statement #3

**Lewis knows that Clara will be a successful musician.**

**TRUE**

FALSE

Statement #4

**When Lewis listens to Clara play the piano, how does he feel?**

SURPRISED

ANGRY

**HAPPY**

SAD

FEAR

DISGUSTED

Statement #5

**If Clara joins the orchestra, Lewis will *not* purchase a ticket to her first concert.**

TRUE

**FALSE**

**High-Uncertainty**

Introduction: This is Lewis. Lewis has a roommate named Clara. Clara plays the piano.

Consistent 1+ Inconsistent 1. Lewis spends most of his evenings listening to Clara play the piano. After several hours of listening to her play, Lewis often gets a headache.

Inconsistent 2. He once went to the mall across the street and bought a pair of noise-cancelling headphones.

Consistent 2. When Clara’s piano needed tuning, Lewis paid to have it done professionally.

Inconsistent 3. During a social get-together at their apartment, Clara played the piano and Lewis wore earplugs during her performance.

Consistent 3. One day, Lewis came across an advertisement to join the local orchestra. Lewis took a copy of the advertisement home and gave it to Clara.

Statement #1

**Lewis believe that Clara is musically talented.**

**TRUE**

FALSE

Statement #2

**Lewis *does not* think that Clara should join the local orchestra.**

TRUE

**FALSE**

Statement #3

**Lewis knows that Clara will be a successful musician.**

**TRUE**

FALSE

Statement #4

**When Lewis listens to Clara play the piano, how does he feel?**

SURPRISED

ANGRY

**HAPPY**

SAD

FEAR

DISGUSTED

Statement #5

**If Clara joins the orchestra, Lewis will *not* purchase a ticket to her first concert.**

TRUE

**FALSE**

**Vignette #2**

**Intermediate-Uncertainty**

Introduction: Meet Sherrie. Sherrie is a second year nursing student at Brown University. Sherrie has four final exams coming up.

Filler 1. Each exam consists of 40 multiple choice questions, 5 short answer questions, and 2 essay questions. Sherrie will have one hour to complete each exam.

Filler 2. Sherrie lives near a local used bookstore. The bookstore opened in the early 1960’s and despite the increased use of e-books on iPads and tablets, the bookstore is still operating.

Consistent 1. Sherrie recently bought an instructional book on the benefits of meditation as a way to relieve stress.

Filler 3. When Sherrie’s hair needed cutting, she always went across the street to her favourite hairdresser for a quick trim.

Filler 4. The next Tuesday, Sherrie and a group of her friends went to the movie theatre to watch a movie. Tickets on Tuesdays at the theatre are half-price.

Consistent 3. On her way home from the theatre, Sherrie saw a class of people stretching in a glass building. The sign in front of the building read “Free Yoga and Meditation Class: All are Welcome”. Sherrie went inside and picked up an information flyer.

Statement #1

**Sherrie does NOT believe that meditation helps relieve stress.**

TRUE

**FALSE**

Statement #2

**Sherrie thinks that meditation has many benefits.**

**TRUE**

FALSE

Statement #3

**Sherrie knows that meditation is good for boosting one’s overall health.**

**TRUE**

FALSE

Statement #4

**If Sherrie was unable to begin meditating, how would she feel?**

SURPRISED

**ANGRY**

HAPPY

**SAD**

FEAR

DISGUSTED

Statement #5

**Sherrie will attend the meditation class.**

**TRUE**

FALSE

**Low-Uncertainty**

Introduction: Meet Sherrie. Sherrie is a second year nursing student at Brown University. Sherrie has four final exams coming up.

Consistent 1. One day, Sherrie saw Oprah Winfrey invite meditation-guru Deepak Chopra on her television talk-show. Sherrie took notes as Deepak gave his five main meditation tips.

Consistent 2. Sherrie recently bought an instructional book on the benefits of meditation as a way to relieve stress.

Consistent 3. After carefully reading every page, Sherrie practiced each of the meditation techniques outlined in the book.

Consistent 4. One evening, Sherrie began writing a weekly blog on the health and stress-relief benefits of meditation.

Consistent 5. The next Tuesday, Sherrie and a group of her friends went to the movie theatre to watch a movie. On their way there, Sherrie shared five meditation tips and 3 useful techniques with her friends.

Consistent 6. On her way home from the theatre, Sherrie saw a class of people stretching in a glass building. The sign in front of the building read “Free Yoga and Meditation Class: All are Welcome”. Sherrie went inside and picked up an information flyer.

Statement #1

**Sherrie does NOT believe that meditation helps relieve stress.**

TRUE

**FALSE**

Statement #2

**Sherrie thinks that meditation has many benefits.**

**TRUE**

FALSE

Statement #3

**Sherrie knows that meditation is good for boosting one’s overall health.**

**TRUE**

FALSE

Statement #4

**If Sherrie was unable to begin meditating, how would she feel?**

SURPRISED

**ANGRY**

HAPPY

**SAD**

FEAR

DISGUSTED

Statement #5

**Sherrie will attend the meditation class.**

**TRUE**

FALSE

**High-Uncertainty**

Introduction: Meet Sherrie. Sherrie is a second year nursing student at Brown University. Sherrie has four final exams coming up.

Consistent 1. One day, Sherrie saw Oprah Winfrey invite meditation-guru Deepak Chopra on her television talk-show. Sherrie took notes as Deepak gave his five main meditation tips.

Inconsistent 1. But the next day in class, she overheard two classmates critiquing the credibility of meditation as a relaxation-technique so during her lunch hour, Sherrie began reading into these critiques.

Consistent 2. Sherrie recently bought an instructional book on the benefits of meditation as a way to relieve stress.

Consistent 3. On her way home from the theatre one night, Sherrie saw a class of people stretching in a glass building. The sign in front of the building read “Free Yoga and Meditation Class: All are Welcome”. Sherrie went inside and picked up an information flyer.

Inconsistent 2. After reading the information flyer, Sherrie learned that classes are held every Tuesday at 8pm. Although Sherrie is available then, she did not schedule the meditation classes in her calendar.

Inconsistent 3. That morning in her nursing classes, Sherrie sat next to the two classmates who critiqued the credibility of meditation.

Statement #1

**Sherrie does NOT believe that meditation helps relieve stress.**

TRUE

**FALSE**

Statement #2

**Sherrie thinks that meditation has many benefits.**

**TRUE**

FALSE

Statement #3

**Sherrie knows that meditation is good for boosting one’s overall health.**

**TRUE**

FALSE

Statement #4

**If Sherrie was unable to begin meditating, how would she feel?**

SURPRISED

**ANGRY**

HAPPY

**SAD**

FEAR

DISGUSTED

Statement #5

**Sherrie will attend the meditation class.**

**TRUE**

FALSE

**Vignette #3**

**Intermediate-Uncertainty**

Introduction: Lydia is a dental assistant. Lydia recently got engaged to Andrew, her high-school sweetheart.

Filler 1. Andrew works as a film director in the city. He studied film at New York University (NYU) and after volunteering for two years at a film agency—Andrew was hired for a full-time position directing short suspense films.

Consistent 1. Most of the time, Lydia keeps the temperature in her and Andrew’s house relatively cool, at about 10 degrees Celsius (or 50 degrees Fahrenheit).

Filler 2. Their wedding is coming up in 6 months and together, Lydia and Andrew are planning their honeymoon vacation.

Filler 3. One of the vacation pamphlets that Andrew brought home suggested Jamaica as a honeymoon destination.

Consistent 2. When Lydia saw this, she looked up the average weather in Jamaica. After learning that the temperature in Jamaica during their honeymoon will reach as high as 33 degrees Celsius (91 degrees Fahrenheit), Lydia raised her eyebrows.

Filler 4. Every night before bed, Lydia reads one chapter of literary fiction.

Statement #1

**Lydia believes that Jamaica is too hot.**

**TRUE**

FALSE

Statement #2

**Lydia thinks that Jamaica is not a good vacation destination for their honeymoon.**

**TRUE**

FALSE

Statement #3

**Lydia knows that if she goes to Jamaica, the heat will bother her.**

**TRUE**

FALSE

Statement #4

**How would Lydia feel if Lydia and Andrew did NOT go to Jamaica for their honeymoon?**

SURPRISED

ANGRY

**HAPPY**

SAD

FEAR

DISGUSTED

Statement #5

**Lydia will not purchase tickets to Jamaica for her and Andrew’s honeymoon.**

**TRUE**

FALSE

**Low-Uncertainty**

Introduction: Lydia is a dental assistant. Lydia recently got engaged to Andrew, her high-school sweetheart.

Consistent 1. Most of the time, Lydia keeps the temperature in her and Andrew’s house relatively cool, at about 10 degrees Celsius (or 50 degrees Fahrenheit).

Consistent 2. In the dental office, Lydia’s colleagues constantly complain about how cold she sets the temperature.

Consistent 3. Even in the winter, Lydia often sleeps without a blanket.

Consistent 4. When discussing possible honeymoon locations, Lydia suggested that she and Andrew consider a winter resort.

Consistent 5. One day at work, Lydia came across advertisement for Jamaica as a honeymoon destination, she looked up the average weather in Jamaica. After learning that the temperature in Jamaica during their honeymoon will reach as high as 33 degrees Celsius (91 degrees Fahrenheit), Lydia raised her eyebrows.

Consistent 6. With a large red marker, Lydia drew a large “X” on the ad for Jamaica.

Statement #1

**Lydia believes that Jamaica is too hot.**

**TRUE**

FALSE

Statement #2

**Lydia thinks that Jamaica is not a good vacation destination for their honeymoon.**

**TRUE**

FALSE

Statement #3

**Lydia knows that if she goes to Jamaica, the heat will bother her.**

**TRUE**

FALSE

Statement #4

**How would Lydia feel if Lydia and Andrew did NOT go to Jamaica for their honeymoon?**

SURPRISED

ANGRY

**HAPPY**

SAD

FEAR

DISGUSTED

Statement #5

**Lydia will not purchase tickets to Jamaica for her and Andrew’s honeymoon.**

**TRUE**

FALSE

**High-Uncertainty**

Introduction: Lydia is a dental assistant. Lydia recently got engaged to Andrew, her high-school sweetheart.

Inconsistent 1. Some of the time, Lydia keeps the temperature in her and Andrew’s house relatively cool, at about 10 degrees Celsius (or 50 degrees Fahrenheit). But other times, Lydia turns up the temperature as hot as 27 degrees Celsius (or 80 degrees Fahrenheit).

Consistent 1. In the dental office, Lydia’s colleagues constantly complain about how cold she sets the temperature.

Consistent 2. Even in the winter, Lydia often sleeps without a blanket.

Inconsistent 2. One day, Lydia visited their local travel agency and picked up a brochure about Jamaica as a honeymoon destination.

Consistent 3. After learning that the temperature in Jamaica during their honeymoon will reach as high as 33 degrees Celsius (91 degrees Fahrenheit), Lydia raised her eyebrows.

Inconsistent 3. The next morning, Lydia purchased a two-piece bathing suit from the mall across the street from her dental office.

Statement #1

**Lydia believes that Jamaica is too hot.**

**TRUE**

FALSE

Statement #2

**Lydia thinks that Jamaica is not a good vacation destination for their honeymoon.**

**TRUE**

FALSE

Statement #3

**Lydia knows that if she goes to Jamaica, the heat will bother her.**

**TRUE**

FALSE

Statement #4

**How would Lydia feel if Lydia and Andrew did NOT go to Jamaica for their honeymoon?**

SURPRISED

ANGRY

**HAPPY**

SAD

FEAR

DISGUSTED

Statement #5

**Lydia will not purchase tickets to Jamaica for her and Andrew’s honeymoon.**

**TRUE**

FALSE

**Vignette #4**

**Intermediate-Uncertainty**

Introduction: Mary is a fourth-year Psychology student at York University.

Filler 1. Mary has two Persian cats—one named Latte, and one named Cappuccino.

Filler 2. Latte has a heart-shaped birth mark on her forehead and wherever she goes, Cappuccino follows her.

Consistent 1. Mary has never purchased an Apple Product in her life. Her laptop is powered by Windows and her cellphone is an Android device.

Consistent 2. When her friends tell her about the newest Apple Product, Mary rolls her eyes.

Filler 3. The day before her final paper for one of her Psychology courses was due, Mary realized that she accidentally left her laptop at her parent’s house—two hours away. With no time left to get her laptop, Mary had to use her roommate’s MacBook (an Apple Product).

Consistent 3. It took Mary nearly two hours to figure out how to use some of the most basic functions of the MacBook.

Statement #1

**Mary believes that Apple Products are overrated.**

**TRUE**

FALSE

Statement #2

**Mary thinks that Apple Products are useful, user-friendly products.**

TRUE

**FALSE**

Statement #3

**Mary knows that if she were to purchase an Apple Product, she would regret it.**

**TRUE**

FALSE

Statement #4

**Now that Mary has to use her roommate's Apple Computer, how does she feel?**

SURPRISED

**ANGRY**

HAPPY

SAD

**AFRAID**

DISGUSTED

Statement #5

**Mary will trade her Android cellphone with an iPhone (an Apple Product).**

TRUE

**FALSE**

**Low-Uncertainty**

Introduction: Mary is a fourth-year Psychology student at York University.

Consistent 1. Mary has never purchased an Apple Product in her life. Her laptop is powered by Windows and her cellphone is an Android device.

Consistent 2. When her friends tell her about the newest Apple Product, Mary rolls her eyes.

Consistent 3. Mary once wrote a blog criticizing Apple products.

Consistent 4. The day before her final paper for one of her Psychology courses was due, Mary realized that she accidentally left her laptop at her parent’s house—two hours away. With no time left to get her laptop, Mary had to use her roommate’s MacBook (an Apple Product). It took Mary nearly two hours to figure out how to use some of the most basic functions of the MacBook.

Consistent 5. Mary remembered how easily she learned how to use programs on her Windows devices.

Consistent 6. After submitting her paper, Mary thanked her roommate for letting her use his MacBook and immediately drove the four hours to her parent’s house to retrieve her Windows laptop.

Statement #1

**Mary believes that Apple Products are overrated.**

**TRUE**

FALSE

Statement #2

**Mary thinks that Apple Products are useful, user-friendly products.**

TRUE

**FALSE**

Statement #3

**Mary knows that if she were to purchase an Apple Product, she would regret it.**

**TRUE**

FALSE

Statement #4

**Now that Mary has to use her roommate's Apple Computer, how does she feel?**

SURPRISED

**ANGRY**

HAPPY

SAD

**AFRAID**

DISGUSTED

Statement #5

**Mary will trade her Android cellphone with an iPhone (an Apple Product).**

TRUE

**FALSE**

**High-Uncertainty**

Introduction: Mary is a fourth-year Psychology student at York University.

Inconsistent 1. Mary frequently visits the Apple electronics store across the street from her apartment.

Consistent 1. But Mary has never in her life purchased an Apple Product. Her laptop is powered by Windows and her cellphone is an Android device.

Consistent 2. When her friends tell her about the newest Apple Product, Mary rolls her eyes.

Inconsistent 2. Mary once watched a half-hour television series about the efficiency and usefulness of Apple electronic products.

Consistent 3. The day before her final paper for one of her Psychology courses was due, Mary realized that she accidentally left her laptop at her parent’s house—two hours away. With no time left to get her laptop, Mary had to use her roommate’s MacBook (an Apple Product). It took Mary nearly two hours to figure out how to use some of the most basic functions of the MacBook.

Inconsistent 3. When she finally figured out how to use her roommate’s MacBook, Mary smiled.

Statement #1

**Mary believes that Apple Products are overrated.**

**TRUE**

FALSE

Statement #2

**Mary thinks that Apple Products are useful, user-friendly products.**

TRUE

**FALSE**

Statement #3

**Mary knows that if she were to purchase an Apple Product, she would regret it.**

**TRUE**

FALSE

Statement #4

**Now that Mary has to use her roommate's Apple Computer, how does she feel?**

SURPRISED

**ANGRY**

HAPPY

SAD

**AFRAID**

DISGUSTED

Statement #5

**Mary will trade her Android cellphone with an iPhone (an Apple Product).**

TRUE

**FALSE**

**Vignette #5**

**Intermediate-Uncertainty**

Introduction: Adrian is software engineer from Boston, Massachusetts. Adrian’s neighbour Ramona works as a fifth-grade school teacher.

Consistent 1. When Adrian runs into Ramona in the elevator, his heart beats faster and his cheeks turn pink.

Consistent 2. Adrian once asked Ramona if she’s single. Ramona replied with a cheerful “yes”.

Filler 1. Adrian has one older sister and one younger brother.

Filler 2. His sister works in the city as a Registered Dietician and his brother plays professional football in the East Coast.

Filler 3. Adrian’s sister is affiliated with the Persons for the Ethical Treatment of Animals (PETA) and she has adopted a vegan lifestyle for nearly a decade.

Filler 4. Adrian tried to be a vegan once but he found it difficult to cut dairy out of his diet.

Statement #1

**Adrian does NOT think that his neighbour Ramona is beautiful.**

TRUE

**FALSE**

Statement #2

**Adrian thinks that he is falling in love with Ramona.**

**TRUE**

FALSE

Statement #3

**Adrian knows that he and Ramona would make a great couple.**

**TRUE**

FALSE

Statement #4

**If Ramona were to kiss Adrian, how would Adrian feel?**

**SURPRISED**

ANGRY

**HAPPY**

SAD

AFRAID

DISGUSTED

Statement #5

**Adrian will ask Ramona out on a romantic date.**

**TRUE**

FALSE

**Low-Uncertainty**

Introduction: Adrian is software engineer from Boston, Massachusetts. Adrian’s neighbour Ramona works as a fifth-grade school teacher.

Consistent 1. When Adrian runs into Ramona in the elevator, his heart beats faster and his cheeks turn pink.

Consistent 2. Adrian once asked Ramona if she’s single. Ramona replied with a cheerful “yes”.

Consistent 3. Ramona bears a striking resemblance to Adrian’s favourite movie star, Natalie Portman.

Consistent 4. Lately, Adrian and Ramona have been spending a considerable amount of time together. Before their get-togethers, Adrian makes sure to look his best.

Consistent 5. Last week, Adrian’s friends persuaded him to go to the local fair and to have their fortunes read by a fortune teller. After reading Adrian’s palm, the fortune teller remarked that his soul-mate is just around the corner.

Consistent 6. Adrian has never met a woman quite like Ramona before.

Statement #1

**Adrian does NOT think that his neighbour Ramona is beautiful.**

TRUE

**FALSE**

Statement #2

**Adrian thinks that he is falling in love with Ramona.**

**TRUE**

FALSE

Statement #3

**Adrian knows that he and Ramona would make a great couple.**

**TRUE**

FALSE

Statement #4

**If Ramona were to kiss Adrian, how would Adrian feel?**

**SURPRISED**

ANGRY

**HAPPY**

SAD

AFRAID

DISGUSTED

Statement #5

**Adrian will ask Ramona out on a romantic date.**

**TRUE**

FALSE

**High-Uncertainty**

Introduction: Adrian is software engineer from Boston, Massachusetts. Adrian’s neighbour Ramona works as a fifth-grade school teacher.

Consistent 1. When Adrian runs into Ramona in the elevator, his heart beats faster and his cheeks turn pink.

Inconsistent 1. Yesterday, Adrian noticed that Ramona cut her hair very short. Adrian has never been attracted to a woman with short hair.

Inconsistent 2. When Adrian’s mother asked him if he’s dating anyone, he replied that he does not have the time for a romantic relationship.

Consistent 2. Adrian once asked Ramona if she’s single. Ramona replied with a cheerful “yes”.

Consistent 3. Lately, Adrian and Ramona have been spending a considerable amount of time together. Before their get-togethers, Adrian makes sure to look his best.

Inconsistent 3. Adrian and Ramona have strikingly different views on a number of political issues. Adrian finds that he has recently found very little in common with Ramona.

Statement #1

**Adrian does NOT think that his neighbour Ramona is beautiful.**

TRUE

**FALSE**

Statement #2

**Adrian thinks that he is falling in love with Ramona.**

**TRUE**

FALSE

Statement #3

**Adrian knows that he and Ramona would make a great couple.**

**TRUE**

FALSE

Statement #4

**If Ramona were to kiss Adrian, how would Adrian feel?**

**SURPRISED**

ANGRY

**HAPPY**

SAD

AFRAID

DISGUSTED

Statement #5

**Adrian will ask Ramona out on a romantic date.**

**TRUE**

FALSE

**Vignette #6**

**Intermediate-Uncertainty**

Introduction: Courtney works for a marketing agency in downtown Toronto, Ontario.

Filler 1. Courtney recently leased a brand new Toyota Matrix. Her previous car was a 2001 Ford Focus. Courtney finds that the Matrix has a much more spacious interior and is very fuel-efficient.

Consistent 1. Last New Year’s Eve, Courtney had a glass of champagne and she felt sick for three consecutive days.

Filler 2. Courtney plays competitive chess every other weekend at the local library.

Filler 3. She recently lost a game to a 12-year-old boy—the youngest player to win a chess game at the library’s biweekly competitions in over 10 years.

Filler 4. Each year, the marketing agency that she works for throws a company dinner during which three employees are chosen at random to win a prize.

Consistent 2. At this year’s company dinner, Courtney won a bottle of white wine. Courtney remembered how much her friend Susan likes white wine. That night, Courtney put the bottle of wine in a paper bag onto which she wrote: “Susan”.

Statement #1

**Courtney believes that alcohol is repulsive.**

**TRUE**

FALSE

Statement #2

**Courtney thinks that drinking alcohol is fun.**

TRUE

**FALSE**

Statement #3

**Courtney knows that alcohol consumption has negative health consequences.**

**TRUE**

FALSE

Statement #4

**If Courtney were to drink an alcoholic drink, how would she feel?**

SURPRISED

ANGRY

HAPPY

SAD

AFRAID

**DISGUSTED**

Statement #5

**Courtney will NOT drink the bottle of wine that she won at the company dinner.**

**TRUE**

FALSE

**Low-Uncertainty**

Introduction: Courtney works for a marketing agency in downtown Toronto, Ontario.

Consistent 1. Last New Year’s Eve, Courtney had a glass of champagne and she felt sick for three consecutive days.

Consistent 2. After this, Courtney added the following statement to her list of New Year’s resolutions: “Don’t drink alcohol”.

Consistent 3. Courtney volunteers her time every other weekend to help raise awareness of Mothers Against Drunk Driving (M.A.D.D), an organization aiming to stop drinking and driving incidences.

Consistent 4. During one of the M.A.D.D events, Courtney gave a talk to high-school students about the health risks associated with drinking alcohol along with tips on how to have fun while sober.

Consistent 5. Each year, the marketing agency that she works for throws a company dinner during which Courtney always drinks a non-alcoholic drink.

Consistent 2. At this year’s company dinner, Courtney won a bottle of white wine. Courtney remembered how much her friend Susan likes white wine. That night, Courtney put the bottle of wine in a paper bag onto which she wrote: “Susan”.

Statement #1

**Courtney believes that alcohol is repulsive.**

**TRUE**

FALSE

Statement #2

**Courtney thinks that drinking alcohol is fun.**

TRUE

**FALSE**

Statement #3

**Courtney knows that alcohol consumption has negative health consequences.**

**TRUE**

FALSE

Statement #4

**If Courtney were to drink an alcoholic drink, how would she feel?**

SURPRISED

ANGRY

HAPPY

SAD

AFRAID

**DISGUSTED**

Statement #5

**Courtney will NOT drink the bottle of wine that she won at the company dinner.**

**TRUE**

FALSE

**High-Uncertainty**

Introduction: Courtney works for a marketing agency in downtown Toronto, Ontario.

Consistent 1. Last New Year’s Eve, Courtney had a glass of champagne and she felt sick for three consecutive days.

Consistent 2. After this, Courtney added the following statement to her list of New Year’s resolutions: “Don’t drink alcohol”.

Inconsistent 1. But after a long day’s work, Courtney usually curls up with a book and a glass of wine.

Inconsistent 2. Her friends call her “keg-stand Courtney” because of her high alcohol tolerance.

Inconsistent 3. Each year, the marketing agency that she works for throws a company dinner during which Courtney always drinks a white wine spritzers.

Consistent 3. At this year’s company dinner, Courtney won a bottle of white wine. Courtney remembered how much her friend Susan likes white wine. That night, Courtney put the bottle of wine in a paper bag onto which she wrote: “Susan”.

Statement #1

**Courtney believes that alcohol is repulsive.**

**TRUE**

FALSE

Statement #2

**Courtney thinks that drinking alcohol is fun.**

TRUE

**FALSE**

Statement #3

**Courtney knows that alcohol consumption has negative health consequences.**

**TRUE**

FALSE

Statement #4

**If Courtney were to drink an alcoholic drink, how would she feel?**

SURPRISED

ANGRY

HAPPY

SAD

AFRAID

**DISGUSTED**

Statement #5

**Courtney will NOT drink the bottle of wine that she won at the company dinner.**

**TRUE**

FALSE

**Vignette #7**

**Intermediate-Uncertainty**

Introduction: Meet Michael. Michael is nearly 6 feet tall—taller than all of his friends. Michael has dark brown hair, blue eyes, and a cluster of freckles on the left side of his face.

Filler 1. One day, Michael’s childhood friend Helena came to visit. Helena has studied the culinary arts for nearly a decade!

Filler 2. Helena showed Michael how to make Moussaka—a Greek dish made with eggplants, ground beef, potato, and cheese.

Filler 3. Michael and Helena overestimated how hungry they were and they had nearly two thirds of Moussaka left over.

Filler 4. In fact, they had so much Moussaka left over, that they gave most of it to their neighbours.

Consistent 1. After applying to several positions around the world, Michael got a starting position as an Immigration Lawyer in one of the most expensive parts of London England. The starting salary for the position is much less than Michael anticipated.

Consistent 2. When Michael saw the average cost of living in London England, his palms began to sweat and his heart raced.

Statement #1

**Michael believes that London England too expensive.**

**TRUE**

FALSE

Statement #2

**Michael does NOT think that the cost of living in London England too high.**

TRUE

**FALSE**

Statement #3

**Michael knows that if he were to move to London England, he would struggle financially.**

**TRUE**

FALSE

Statement #4

**Now that Michael might move to London, England, how does he feel when he thinks about his cost of living?**

SURPRISED

ANGRY

HAPPY

SAD

**AFRAID**

DISGUSTED

Statement #5

**If Michael accepts the position, he will apply for a bank loan to help him with his cost of living in London, England.**

**TRUE**

FALSE

**Low-Uncertainty**

Introduction: Meet Michael. Michael is nearly 6 feet tall—taller than all of his friends. Michael has dark brown hair, blue eyes, and a cluster of freckles on the left side of his face.

Consistent 1. Growing up, Michael’s parents were unable to fund his education so he applied for a student loan to help him with the cost of Law School. Michael does not have any assets or any inheritance of any kind.

Consistent 2. After applying to several positions around the world, Michael got a starting position as an Immigration Lawyer in one of the most expensive parts of London England. The starting salary for the position is much less than Michael anticipated.

Consistent 3. When Michael saw the average cost of living in London England, his palms began to sweat and his heart raced.

Consistent 4. That night, Michael Googled “how to get approved for a bank loan” before going to bed.

Consistent 5. Michael’s childhood friend Helena used to live in London England and she told Michael that she sold her car to make ends meet.

Consistent 6. When Michael looked up the price of food in London England and he felt a twisting pain at the pit of his stomach.

Statement #1

**Michael believes that London England too expensive.**

**TRUE**

FALSE

Statement #2

**Michael does NOT think that the cost of living in London England too high.**

TRUE

**FALSE**

Statement #3

**Michael knows that if he were to move to London England, he would struggle financially.**

**TRUE**

FALSE

Statement #4

**Now that Michael might move to London, England, how does he feel when he thinks about his cost of living?**

SURPRISED

ANGRY

HAPPY

SAD

**AFRAID**

DISGUSTED

Statement #5

**If Michael accepts the position, he will apply for a bank loan to help him with his cost of living in London, England.**

**TRUE**

FALSE

**High-Uncertainty**

Introduction: Meet Michael. Michael is nearly 6 feet tall—taller than all of his friends. Michael has dark brown hair, blue eyes, and a cluster of freckles on the left side of his face.

Consistent 1. Growing up, Michael’s parents were unable to fund his education so he applied for a student loan to help him with the cost of Law School. Michael does not have any assets or any inheritance of any kind.

Consistent 2. After applying to several positions around the world, Michael got a starting position as an Immigration Lawyer in one of the most expensive parts of London England. The starting salary for the position is much less than Michael anticipated.

Consistent 3. When Michael saw the average cost of living in London England, his palms began to sweat and his heart raced.

Inconsistent 1. Michael’s childhood friend Helena used to live in London England and she told Michael that the cost of living there is actually quite low.

Inconsistent 2. Helena’s salary was much less than Michaels and she remarked that she was able to rent a reasonably-sized apartment without any trouble.

Inconsistent 3. After purchasing a flat-screen television, Michael smiled when he saw how much money he had leftover.

Statement #1

**Michael believes that London England too expensive.**

**TRUE**

FALSE

Statement #2

**Michael does NOT think that the cost of living in London England too high.**

TRUE

**FALSE**

Statement #3

**Michael knows that if he were to move to London England, he would struggle financially.**

**TRUE**

FALSE

Statement #4

**Now that Michael might move to London, England, how does he feel when he thinks about his cost of living?**

SURPRISED

ANGRY

HAPPY

SAD

**AFRAID**

DISGUSTED

Statement #5

**If Michael accepts the position, he will apply for a bank loan to help him with his cost of living in London, England.**

**TRUE**

FALSE

**Vignette #8**

**Intermediate-Uncertainty**

Introduction. This is Clarissa. Clarissa’s favourite colour is turquoise and the majority of her clothes and accessories are turquoise.

Consistent 1. Clarissa’s neighbours are a gay couple who recently adopted a little boy from Romania. When Clarissa sees her gay neighbours playing with their son, she shakes her head and rolls her eyes.

Consistent 2. Every year, the gay parade is held two blocks away from Clarissa’s house. Clarissa’s friend James lives far away from the location of the gay parade and so, Clarissa stays at James’s house during the gay parade.

Filler 1. James has a parrot named Prince. Prince has a beautiful coat of vibrant orange and green feathers.

Filler 2. Prince repeats everything that James says verbatim. When James was learning to speak Spanish, Prince began muttering words in Spanish.

Filler 3. Sometimes, James listens to music while he cooks dinner and Prince dances along to the beat of the song.

Filler 4. James once videotaped Prince dancing and uploaded onto YouTube. Prince’s video has over 200 “likes” so far.

Statement #1

**Clarissa believes that marriage is only between a man and a woman.**

TRUE

**FALSE**

Statement #2

**Clarissa thinks that every country should strive to promote marriage equality, meaning that same-sex marriage should be legalized and celebrated across the world.**

TRUE

**FALSE**

Statement #3

**Clarissa knows that gay and lesbian couples deserve the right to celebrate their love for one another through marriage**

TRUE

**FALSE**

Statement #4

**If same-sex marriage were legalized in every country across the world, Clarissa would feel the following.**

SURPRISED

**ANGRY**

HAPPY

SAD

AFRAID

DISGUSTED

Statement #5

**Clarissa will NOT attend this year’s gay parade.**

**TRUE**

FALSE

**Low-Uncertainty**

Introduction. This is Clarissa. Clarissa’s favourite colour is turquoise and the majority of her clothes and accessories are turquoise.

Consistent 1. Clarissa once wrote a persuasive blog against same-sex marriage.

Consistent 2. Clarissa’s neighbours are a gay couple who recently adopted a little boy from Romania. When Clarissa sees her gay neighbours playing with their son, she shakes her head and rolls her eyes.

Consistent 3. Every year, the gay parade is held two blocks away from Clarissa’s house. Clarissa’s friend James lives far away from the location of the gay parade and so, Clarissa stays at James’s house during the gay parade.

Consistent 4. One day, Clarissa was watching television and Ellen DeGeneres—an openly gay talk-show host—was telling her usual light-hearted jokes. Clarissa immediately changed the channel.

Consistent 5. Last summer, one of Clarissa’s colleagues Tara married her girlfriend Virginia. When Clarissa got her invitation in the mail, she checked off the box that read “Regretfully Decline”.

Consistent 6. At a local garage sale, Clarissa found a cozy sweater at a bargain. But after reading the slogan “Gay and Proud” on the front pocket, Clarissa put the sweater down and purchased a pair of gloves instead.

Statement #1

**Clarissa believes that marriage is only between a man and a woman.**

TRUE

**FALSE**

Statement #2

**Clarissa thinks that every country should strive to promote marriage equality, meaning that same-sex marriage should be legalized and celebrated across the world.**

TRUE

**FALSE**

Statement #3

**Clarissa knows that gay and lesbian couples deserve the right to celebrate their love for one another through marriage**

TRUE

**FALSE**

Statement #4

**If same-sex marriage were legalized in every country across the world, Clarissa would feel the following.**

SURPRISED

**ANGRY**

HAPPY

SAD

AFRAID

DISGUSTED

Statement #5

**Clarissa will NOT attend this year’s gay parade.**

**TRUE**

FALSE

**High-Uncertainty**

Introduction. This is Clarissa. Clarissa’s favourite colour is turquoise and the majority of her clothes and accessories are turquoise.

Consistent 1. Clarissa once wrote a persuasive essay against same-sex marriage.

Inconsistent 1. She wrote the essay as part of a course project. Clarissa did not choose the topic.

Consistent 2. Clarissa’s neighbours are a gay couple who recently adopted a little boy from Romania. When Clarissa sees her gay neighbours playing with their son, she shakes her head and rolls her eyes.

Inconsistent 2. When Clarissa comes home from work, she usually makes her dinner and watches Ellen DeGeneres—an openly gay talk-show host tell jokes on television.

Consistent 3. Every year, the gay parade is held two blocks away from Clarissa’s house. Clarissa’s friend James lives far away from the location of the gay parade and so, Clarissa stays at James’s house during the gay parade.

Inconsistent 3. When Clarissa learned that in some countries, gay and lesbian couples are killed, she cried.

Statement #1

**Clarissa believes that marriage is only between a man and a woman.**

TRUE

**FALSE**

Statement #2

**Clarissa thinks that every country should strive to promote marriage equality, meaning that same-sex marriage should be legalized and celebrated across the world.**

TRUE

**FALSE**

Statement #3

**Clarissa knows that gay and lesbian couples deserve the right to celebrate their love for one another through marriage**

TRUE

**FALSE**

Statement #4

**If same-sex marriage were legalized in every country across the world, Clarissa would feel the following.**

SURPRISED

**ANGRY**

HAPPY

SAD

AFRAID

DISGUSTED

Statement #5

**Clarissa will NOT attend this year’s gay parade.**

**TRUE**

FALSE

**Vignette #9**

**Intermediate-Uncertainty**

Introduction. Meet Emanuel. Emanuel has a keen interest in painting. He once enrolled in a local painting competition and won the grand prize of $500.00.

Filler 1. Emanuel purchases his painting supplies from a wholesale supplier that is located nearly 40 minutes away from Emanuel’s house.

Consistent 1. Emanuel’s friend Lindsay often hosts parties at her house. When Emanuel goes to Lindsay’s house, he is welcomed with open arms, fresh fruit, and a delicious dinner.

Filler 2. While painting in his house one night, Emanuel thought he heard music playing from down the street.

Filler 3. When he went to see where the sound was coming from, Emanuel learned that his neighbour’s 5-year-old daughter was playing the piano.

Consistent 2. The next day, Emanuel ate a light lunch because he was invited to Lindsay’s house for dinner that evening.

Filler 4. On the car ride to Lindsay’s house, Emanuel couldn’t help but notice the beautiful starry night sky.

Statement #1

**Emanuel does NOT believe that Lindsay is hospitable.**

TRUE

**FALSE**

Statement #2

**Emanuel thinks that Lindsay is hospitable.**

**TRUE**

FALSE

Statement #3

**Emanuel knows that if he were to go to Lindsay's house, she would be a warm and inviting host.**

**TRUE**

FALSE

Statement #4

**If Emanuel were to go to Lindsay's house and she offered him no food or beverages all night, how would Emanuel feel about Lindsay's behaviour?**

**SURPRISED**

ANGRY

HAPPY

SAD

AFRAID

DISGUSTED

Statement #5

**When asked what kind of host Lindsay is, Emanuel will reply, "hospitable".**

**TRUE**

FALSE

**Low-Uncertainty**

Introduction. Meet Emanuel. Emanuel has a keen interest in painting. He once enrolled in a local painting competition and won the grand prize of $500.00.

Consistent 1. Emanuel’s friend Lindsay often hosts parties at her house. When Emanuel goes to Lindsay’s house, he is welcomed with open arms, fresh fruit, and a delicious dinner.

Consistent 2. The guests at Lindsay’s parties often play a game in which an adjective is said and everyone points to the person most likely to fit that description. When the word “hospitable host” came up one night, Emanuel immediately pointed at Lindsay.

Consistent 3. One day, Emanuel ate a light lunch because he was invited to Lindsay’s house for dinner that evening.

Consistent 4. When Emanuel and his friends arrived at Lindsay’s house, Lindsay was her usual self. She opened the door with a smile as her guests came in and took each and every one of their coats.

Consistent 5. While her guests were waiting for dinner to be served, Lindsay offered each guest a drink along with an appetizer of their choice.

Consistent 6. One of Lindsay’s party traditions is to give each guest a small gift when they leave and tonight was no exception. Lindsay gave each guest a bouquet of fresh fruit designed to resemble an arrangement of flowers.

Statement #1

**Emanuel does NOT believe that Lindsay is hospitable.**

TRUE

**FALSE**

Statement #2

**Emanuel thinks that Lindsay is hospitable.**

**TRUE**

FALSE

Statement #3

**Emanuel knows that if he were to go to Lindsay's house, she would be a warm and inviting host.**

**TRUE**

FALSE

Statement #4

**If Emanuel were to go to Lindsay's house and she offered him no food or beverages all night, how would Emanuel feel about Lindsay's behaviour?**

**SURPRISED**

ANGRY

HAPPY

SAD

AFRAID

DISGUSTED

Statement #5

**When asked what kind of host Lindsay is, Emanuel will reply, "hospitable".**

**TRUE**

FALSE

**High-Uncertainty**

Introduction. Meet Emanuel. Emanuel has a keen interest in painting. He once enrolled in a local painting competition and won the grand prize of $500.00.

Inconsistent 1. Emanuel’s friend Lindsay often hosts parties at her house. When Emanuel goes to Lindsay’s house, he must hold on to his jacket all night and if he’s lucky, Lindsay might offer Emanuel and the rest of Lindsay’s guests a glass of water.

Consistent 1. The guests at Lindsay’s parties often play a game in which an adjective is said and everyone points to the person most likely to fit that description. When the word “hospitable host” came up one night, Emanuel immediately pointed at Lindsay.

Inconsistent 2. But then, everyone immediately burst into laughter.

Inconsistent 3. Lindsay has never served her guests a proper meal during any of her parties.

Consistent 2. One day, Emanuel ate a light lunch because he was invited to Lindsay’s house for dinner that evening.

Consistent 3. One of Lindsay’s party traditions is to give each guest a small gift when they leave and tonight was no exception. Lindsay gave each guest a bouquet of fresh fruit designed to resemble an arrangement of flowers.

Statement #1

**Emanuel does NOT believe that Lindsay is hospitable.**

TRUE

**FALSE**

Statement #2

**Emanuel thinks that Lindsay is hospitable.**

**TRUE**

FALSE

Statement #3

**Emanuel knows that if he were to go to Lindsay's house, she would be a warm and inviting host.**

**TRUE**

FALSE

Statement #4

**If Emanuel were to go to Lindsay's house and she offered him no food or beverages all night, how would Emanuel feel about Lindsay's behaviour?**

**SURPRISED**

ANGRY

HAPPY

SAD

AFRAID

DISGUSTED

Statement #5

**When asked what kind of host Lindsay is, Emanuel will reply, "hospitable".**

**TRUE**

FALSE

**Vignette #10**

**Intermediate-Uncertainty**

Introduction. Dana is fourth-year student at the University of Alberta. During the winter months in particular, Alberta can get especially cold.

Consistent 1. Both of Dana’s parents are medical doctors: her mother is a cardiologist and her father is a family doctor. But on “take your kids to work day”, Dana never shadows her mother or father in the medical field.

Consistent 2. Last week, Dana’s friend fell off of his bicycle and he needed a small number of stitches on his left shoulder. During the procedure, Dana tightly shut her eyes.

Filler 1. One of Dana’s professors, Dr. Hurley has a strong sense of humour. When Dr. Hurley teaches a class, the room fills with laughter.

Filler 2. Dr. Hurley once dressed up as a giant chocolate chip cookie after losing a bet with one of his students.

Filler 3. In his teacher reviews, Dr. Hurley often gets comments like “hilarious!” and “best professor ever!”

Filler 4. Dr. Hurley was married twice and from each marriage, he has two children—making him a father of four children in total.

Statement #1

**Dana believes that she would make a great medical doctor.**

TRUE

**FALSE**

Statement #2

**Dana thinks that she should study medicine in medical school.**

TRUE

**FALSE**

Statement #3

**Dana knows that if she were to work as a medical doctor, she would be terrible.**

**TRUE**

FALSE

Statement #4

**If Dana was shown a bleeding patient on the operating table, how would she feel?**

SURPRISED

ANGRY

HAPPY

SAD

**AFRAID**

**DISGUSTED**

Statement #5

**Dana will NOT apply to medical school.**

**TRUE**

FALSE

**Low-Uncertainty**

Introduction. Dana is fourth-year student at the University of Alberta. During the winter months in particular, Alberta can get especially cold.

Consistent 1. Both of Dana’s parents are medical doctors: her mother is a cardiologist and her father is a family doctor. But on “take your kids to work day”, Dana never shadows her mother or father in the medical field.

Consistent 2. Last week, Dana’s friend fell off of his bicycle and he needed a small number of stitches on his left shoulder. During the procedure, Dana tightly shut her eyes.

Consistent 3. When asked “what is your biggest fear?” Dana often replies “blood!”

Consistent 4. Earlier this month, Dana purchased a set of books designed to help prepare students for Law School.

Consistent 5. Dana once remarked that a great doctor is both fearless and passionate about medicine. Dana has never described herself in those terms.

Consistent 6. The due date for medical school applications is January 15^th^ and the due date for law school applications is March 1^st^. In Dana’s calendar, March 1^st^ is highlighted by January 15^th^ is left blank.

Statement #1

**Dana believes that she would make a great medical doctor.**

TRUE

**FALSE**

Statement #2

**Dana thinks that she should study medicine in medical school.**

TRUE

**FALSE**

Statement #3

**Dana knows that if she were to work as a medical doctor, she would be terrible.**

**TRUE**

FALSE

Statement #4

**If Dana was shown a bleeding patient on the operating table, how would she feel?**

SURPRISED

ANGRY

HAPPY

SAD

**AFRAID**

**DISGUSTED**

Statement #5

**Dana will NOT apply to medical school.**

**TRUE**

FALSE

**High-Uncertainty**

Introduction. Dana is fourth-year student at the University of Alberta. During the winter months in particular, Alberta can get especially cold.

Consistent 1. Both of Dana’s parents are medical doctors: her mother is a cardiologist and her father is a family doctor. But on “take your kids to work day”, Dana never shadows her mother or father in the medical field.

Inconsistent 1. Earlier this month, Dana purchased a set of books designed to help prepare students for Medical School.

Inconsistent 2. Dana’s favourite television show is “Grey’s Anatomy”—a drama pertaining to the lives of medical interns.

Consistent 2. Last week, Dana’s friend fell off of his bicycle and he needed a small number of stitches on his left shoulder. During the procedure, Dana tightly shut her eyes.

Consistent 3. Dana once remarked that a great doctor is both fearless and passionate about medicine. Dana has never described herself in those terms.

Inconsistent 3. The due date for medical school applications is January 15^th^. In Dana’s calendar, January 15^th^ is highlighted.

Statement #1

**Dana believes that she would make a great medical doctor.**

TRUE

**FALSE**

Statement #2

**Dana thinks that she should study medicine in medical school.**

TRUE

**FALSE**

Statement #3

**Dana knows that if she were to work as a medical doctor, she would be terrible.**

**TRUE**

FALSE

Statement #4

**If Dana was shown a bleeding patient on the operating table, how would she feel?**

SURPRISED

ANGRY

HAPPY

SAD

**AFRAID**

**DISGUSTED**

Statement #5

**Dana will NOT apply to medical school.**

**TRUE**

FALSE

**Vignette #11**

**Intermediate-Uncertainty**

Introduction. Isabelle has long blonde hair and dark brown eyes. On Friday’s, Isabelle volunteers her time to help a local charity for low-income families.

Filler 1. The charity was originally called “Neighbours Lending a Helping Hand”, but the acronym N.L.H.H was difficult to remember. Therefore, the charity name was changed to “Our Ambition to Heal”, or “OATH” for short.

Filler 2. Isabelle learned about Oath’s initiative after listening to her niece Crystal present a school project on community volunteering opportunities.

Consistent 1. Isabelle’s coworkers always try to persuade her to have something other than sushi for lunch but nearly every day, Isabelle buys a few rolls of sushi for lunch.

Filler 3. On the day before the start of the winter holidays, Isabelle and her colleagues celebrate with dinner at a high-end restaurant.

Consistent 2. This year, the restaurant they went to had a Intermediate-Uncertainty menu with North American dishes as well as a sushi menu. Isabelle didn’t even look at the Intermediate-Uncertainty menu.

Filler 4. After dinner, Isabelle and her colleagues planned this year’s company New Year’s Eve party.

Statement #1

**Isabelle believes that eating sushi is disgusting.**

TRUE

**FALSE**

Statement #2

**Isabelle thinks that sushi is delicious.**

**TRUE**

FALSE

Statement #3

**Isabelle knows that if she were to eat sushi for lunch, she would enjoy it.**

**TRUE**

FALSE

Statement #4

**If Isabelle were to win a free sushi lunch, how would she feel?**

SURPRISED

ANGRY

**HAPPY**

SAD

AFRAID

DISGUSTED

Statement #5

**Isabelle will choose a dish from the sushi menu at the restaurant.**

**TRUE**

FALSE

**Low-Uncertainty**

Introduction. Isabelle has long blonde hair and dark brown eyes. On Friday’s, Isabelle volunteers her time to help a local charity for low-income families.

Consistent 1. Isabelle’s coworkers always try to persuade her to have something other than sushi for lunch but nearly every day, Isabelle buys a few rolls of sushi for lunch.

Consistent 2. One day, Isabelle and her colleagues went to a high-end restaurant to celebrate the start of the winter holidays. The restaurant had a Intermediate-Uncertainty menu with North American dishes as well as a sushi menu. Isabelle didn’t even look at the Intermediate-Uncertainty menu.

Consistent 3. When one of Isabelle’s colleagues remarked that eating sushi is disgusting, Isabelle frowned.

Consistent 4. Growing up, Isabelle had the opportunity to visit Japan and eat authentic sushi. Although the sushi in North America does not compare to what she had in Japan—Isabelle continues to order sushi whenever it is available.

Consistent 5. Isabelle once watched a 7-part documentary on the history and origins of sushi. As she watched the chefs in the documentary make each roll, Isabelle’s mouth watered.

Consistent 6. The day after the company dinner at the restaurant, Isabelle googled “Sushi Delivery” and ordered 3 rolls of sushi while watching television.

Statement #1

**Isabelle believes that eating sushi is disgusting.**

TRUE

**FALSE**

Statement #2

**Isabelle thinks that sushi is delicious.**

**TRUE**

FALSE

Statement #3

**Isabelle knows that if she were to eat sushi for lunch, she would enjoy it.**

**TRUE**

FALSE

Statement #4

**If Isabelle were to win a free sushi lunch, how would she feel?**

SURPRISED

ANGRY

**HAPPY**

SAD

AFRAID

DISGUSTED

Statement #5

**Isabelle will choose a dish from the sushi menu at the restaurant.**

**TRUE**

FALSE

**High-Uncertainty**

Introduction. Isabelle has long blonde hair and dark brown eyes. On Friday’s, Isabelle volunteers her time to help a local charity for low-income families.

Consistent 1. Isabelle’s coworkers always try to persuade her to have something other than sushi for lunch but nearly every day, Isabelle buys a few rolls of sushi for lunch.

Inconsistent 1. Although on a few occasions, Isabelle has felt sick after eating sushi for lunch.

Inconsistent 2. Isabelle once went on a romantic date to a sushi restaurant and asked the waiter if they have “fries or pasta”.

Consistent 2. One day, Isabelle and her colleagues went to a high-end restaurant to celebrate the start of the winter holidays. The restaurant had a Intermediate-Uncertainty menu with North American dishes as well as a sushi menu. Isabelle didn’t even look at the Intermediate-Uncertainty menu.

Inconsistent 3. But after carefully scanning the options on the Intermediate-Uncertainty menu, Isabelle took a look at the sushi menu.

Consistent 3. When one of Isabelle’s colleagues remarked that eating sushi is disgusting, Isabelle frowned.

Statement #1

**Isabelle believes that eating sushi is disgusting.**

TRUE

**FALSE**

Statement #2

**Isabelle thinks that sushi is delicious.**

**TRUE**

FALSE

Statement #3

**Isabelle knows that if she were to eat sushi for lunch, she would enjoy it.**

**TRUE**

FALSE

Statement #4

**If Isabelle were to win a free sushi lunch, how would she feel?**

SURPRISED

ANGRY

**HAPPY**

SAD

AFRAID

DISGUSTED

Statement #5

**Isabelle will choose a dish from the sushi menu at the restaurant.**

**TRUE**

FALSE

**Vignette #12**

**Intermediate-Uncertainty**

Introduction. Jared is an 8-year-old boy. Jared attends Eagle Hawks Elementary school.

Filler 1. Jared plays competitive hockey for the city of Hamilton, Ontario. Jared’s father is the coach on the hockey team.

Consistent 1. In his free time, Jared reads ghost stories about spirits who live in haunted houses.

Filler 2. Mrs. Truman, Jared’s next-door-neighbour, is an 85-year-old woman who devoted nearly 40 years of her life to a career as a nurse practitioner.

Consistent 2. One day, while playing road hockey on his street with his friends, Jared spotted a shadow in one of the windows of Mrs. Truman’s house. He shouted “ghost!” and together, Jared and his friends all ran home.

Filler 3. At home, Jared’s mother was making home-made macaroni and cheese with a side of potato wedges—her specialty.

Filler 4. The next morning, Jared and his parents walked the two blocks down to the local farmer’s market to buy two jars of their famous cherry and strawberry jam.

Statement #1

**Jared believes that there are ghosts living in Mrs. Truman's house.**

**TRUE**

FALSE

Statement #2

**Jared does NOT think that Mrs. Truman's house is haunted.**

TRUE

**FALSE**

Statement #3

**Jared knows that there are spirits living in Mrs. Truman's house.**

**TRUE**

FALSE

Statement #4

**If Jared were to go inside Mrs. Truman's house, how would he feel?**

SURPRISED

ANGRY

HAPPY

SAD

**AFRAID**

DISGUSTED

Statement #5

**Whenever possible, Jared will avoid Mrs. Truman's house.**

**TRUE**

FALSE

**Low-Uncertainty**

Introduction. Jared is an 8-year-old boy. Jared attends Eagle Hawks Elementary school.

Consistent 1. In his free time, Jared reads ghost stories about spirits who live in haunted houses.

Consistent 2. Mrs. Truman, Jared’s next-door-neighbour, is an 85-year-old woman who once invited Jared over for piano lessons. Despite the fact that Mrs. Truman is a sweet and pleasant old woman, Jared refused to go to her house.

Consistent 3. One day, while playing road hockey on his street with his friends, Jared spotted a shadow in one of the windows of Mrs. Truman’s house. He shouted “ghost!” and together, Jared and his friends all ran home.

Consistent 4. He once heard voices coming from Mrs. Truman’s house while she was away at a doctor’s appointment.

Consistent 5. When Jared told his parents, they dismissed what he heard so instead, Jared told his friends at school.

Consistent 6. Jared has never gone inside Mrs. Truman’s house. Even on Halloween, he refused to go ‘trick or treating’ at Mrs. Truman’s house.

Statement #1

**Jared believes that there are ghosts living in Mrs. Truman's house.**

**TRUE**

FALSE

Statement #2

**Jared does NOT think that Mrs. Truman's house is haunted.**

TRUE

**FALSE**

Statement #3

**Jared knows that there are spirits living in Mrs. Truman's house.**

**TRUE**

FALSE

Statement #4

**If Jared were to go inside Mrs. Truman's house, how would he feel?**

SURPRISED

ANGRY

HAPPY

SAD

**AFRAID**

DISGUSTED

Statement #5

**Whenever possible, Jared will avoid Mrs. Truman’s house.**

**TRUE**

FALSE

**High-Uncertainty**

Introduction. Jared is an 8-year-old boy. Jared attends Eagle Hawks Elementary school.

Consistent 1. In his free time, Jared reads ghost stories about spirits who live in haunted houses.

Inconsistent 1. But he usually laughs while reading these books.

Inconsistent 2. When Jared’s little sister told him that she was scared of the ghost under her bed, Jared smiled and said “no, silly! Ghosts aren’t real!”

Consistent 2. Mrs. Truman, Jared’s next-door-neighbour, is an 85-year-old woman who once invited Jared over for piano lessons. Despite the fact that Mrs. Truman is a sweet and pleasant old woman, Jared refused to go to her house.

Inconsistent 3. He preferred to play video games instead and this year, Jared has done exceptionally well in school so his parents let him stay inside instead.

Consistent 3. One day, while playing road hockey on his street with his friends, Jared spotted a shadow in one of the windows of Mrs. Truman’s house. He shouted “ghost!” and together, Jared and his friends all ran home.

Consistent 4. He once heard voices coming from Mrs. Truman’s house while she was away at a doctor’s appointment.

Statement #1

**Jared believes that there are ghosts living in Mrs. Truman's house.**

**TRUE**

FALSE

Statement #2

**Jared does NOT think that Mrs. Truman's house is haunted.**

TRUE

**FALSE**

Statement #3

**Jared knows that there are spirits living in Mrs. Truman's house.**

**TRUE**

FALSE

Statement #4

**If Jared were to go inside Mrs. Truman's house, how would he feel?**

SURPRISED

ANGRY

HAPPY

SAD

**AFRAID**

DISGUSTED

Statement #5

**Whenever possible, Jared will avoid Mrs. Truman's house.**

**TRUE**

FALSE

**Vignette #13**

**Intermediate-Uncertainty**

Introduction. Dr. Watkins is a senior professor of Economics at Carnegie Mellon University in Pittsburgh, Pennsylvania. Three years ago, Dr. Watkins took on PhD student Timothy Jensen. Timothy plans on writing a book chapter for this year’s Introductory Economics textbook.

Filler 1. Timothy’s parents live in Miami Florida. It would take nearly 20 hours to drive from his house to visit his parents and so, Timothy usually catches the 3-hour plane directly to Miami to see his parents.

Consistent 1. While proof-reading one of Timothy’s papers, Dr. Watkins once sighed and asked his wife for an Advil.

Consistent 2. When he finally read through the entire paper, Dr. Watkins had nearly 4 full pages of feedback for Timothy ranging from comments like “unclear writing” to “poor academic writing skills—please see me”.

Filler 2. When Timothy got the email from Dr. Watkins with revisions to his paper, he was watching a baseball game with his girlfriend Madalyn.

Filler 3. Madalyn likes to watch baseball almost as much as she likes to play baseball.

Filler 4. Timothy and Madalyn have been dating for nearly half a decade.

Statement #1

**Dr. Watkins believes that Timothy's writing needs a significant amount of improvement.**

**TRUE**

FALSE

Statement #2

**Dr. Watkins does NOT think that Timothy is a skillful academic writer.**

**TRUE**

FALSE

Statement #3

**Dr. Watkins knows that Timothy's book chapter will not get published in this year's Introductory Economics textbook.**

**TRUE**

FALSE

Statement #4

**If Timothy's chapter was published in this year's Introductory Economics textbook, how would Dr. Watkins feel?**

**SURPRISED**

ANGRY

HAPPY

SAD

AFRAID

DISGUSTED

Statement #5

**Dr. Watkins will NOT take writing advice from Timothy.**

**TRUE**

FALSE

**Low-Uncertainty**

Introduction. Dr. Watkins is a senior professor of Economics at Carnegie Mellon University in Pittsburgh, Pennsylvania. Three years ago, Dr. Watkins took on PhD student Timothy Jensen. Timothy plans on writing a book chapter for this year’s Introductory Economics textbook.

Consistent 1. While proof-reading one of Timothy’s papers, Dr. Watkins once sighed and asked his wife for an Advil.

Consistent 2. When he finally read through the entire paper, Dr. Watkins had nearly 4 full pages of feedback for Timothy ranging from comments like “unclear writing” to “poor academic writing skills—please see me”.

Consistent 3. Dr. Watkins once gave a lecture on how difficult it is to publish a book chapter in an Introductory Economics textbook.

Consistent 4. In his lecture, he noted that the editors of the textbook have very high standards regarding writing style and clarity of academic writing.

Consistent 5. Dr. Watkins once recommended that Timothy attend a “Basic Writing” workshop but unfortunately, Timothy was out of town during the workshop and could not attend.

Consistent 6. During his evaluation of Timothy’s performance last year, Dr. Watkins checked off the box titled “Needs Improvement” for the writing portion of Timothy’s performance.

Statement #1

**Dr. Watkins believes that Timothy's writing needs a significant amount of improvement.**

**TRUE**

FALSE

Statement #2

**Dr. Watkins does NOT think that Timothy is a skillful academic writer.**

**TRUE**

FALSE

Statement #3

**Dr. Watkins knows that Timothy's book chapter will not get published in this year's Introductory Economics textbook.**

**TRUE**

FALSE

Statement #4

**If Timothy's chapter was published in this year's Introductory Economics textbook, how would Dr. Watkins feel?**

**SURPRISED**

ANGRY

HAPPY

SAD

AFRAID

DISGUSTED

Statement #5

**Dr. Watkins will NOT take writing advice from Timothy.**

**TRUE**

FALSE

**High-Uncertainty**

Introduction. Dr. Watkins is a senior professor of Economics at Carnegie Mellon University in Pittsburgh, Pennsylvania. Three years ago, Dr. Watkins took on PhD student Timothy Jensen. Timothy plans on writing a book chapter for this year’s Introductory Economics textbook.

Inconsistent 1. Dr. Watkins once recommended that Timothy teach a workshop on academic writing and when Timothy did, Dr. Watkins attended the workshop and took notes.

Inconsistent 2. When introducing Timothy to his colleagues, Dr. Watkins once praised Timothy for his academic writing skills.

Inconsistent 3. During his evaluation of Timothy’s performance last year, Dr. Watkins checked off the box titled “Excellent” for the writing portion of Timothy’s performance.

Consistent 1. While proof-reading one of Timothy’s papers, Dr. Watkins once sighed and asked his wife for an Advil.

Consistent 2. When he finally read through the entire paper, Dr. Watkins had nearly 4 full pages of feedback for Timothy ranging from comments like “unclear writing” to “poor academic writing skills—please see me”.

Consistent 3. Dr. Watkins once gave a lecture on how difficult it is to publish a book chapter in an Introductory Economics textbook.

Statement #1

**Dr. Watkins believes that Timothy's writing needs a significant amount of improvement.**

**TRUE**

FALSE

Statement #2

**Dr. Watkins does NOT think that Timothy is a skillful academic writer.**

**TRUE**

FALSE

Statement #3

**Dr. Watkins knows that Timothy's book chapter will not get published in this year's Introductory Economics textbook.**

**TRUE**

FALSE

Statement #4

**If Timothy's chapter was published in this year's Introductory Economics textbook, how would Dr. Watkins feel?**

**SURPRISED**

ANGRY

HAPPY

SAD

AFRAID

DISGUSTED

Statement #5

**Dr. Watkins will NOT take writing advice from Timothy.**

**TRUE**

FALSE

**Vignette #14**

**Intermediate-Uncertainty**

Introduction. Meet Sylvia. Sylvia is a 23-year-old administrative assistant at a popular car insurance company. Sylvia’s long-time boyfriend Sean recently proposed to her with a 2-carat princess-cut engagement ring.

Consistent 1. Last Thursday, Sylvia drove 3 and a half hours to visit her college roommate Bethany. Sylvia and Bethany got their nails done on Friday morning at a local nail salon in Bethany’s town. Sylvia requested a hand-exfoliation massage so she removed her engagement ring and placed it on the counter at the salon.

Filler 1. During the drive back from Bethany’s house, Sylvia listened to her favourite radio station: “I-Heart-Radio”.

Filler 2. She learned that the winter Olympics will be held in Canada this year and when the radio began its “Sports” segment, Sylvia changed the channel.

Consistent 2. As she reached for the dial, she noticed how clean her hands look and immediately, it occurred to her that she’s missing her engagement ring! In a panic, Sylvia pulled over, called Bethany, and asked for the nail salon’s telephone number.

Filler 3. As she waited for Bethany to answer her cellphone, Sylvia noticed a red pick-up truck with a group of carefree teenagers drive by.

Filler 4. In the front seat was a lively brunette wearing dark sunglasses and singing as her hair danced around her face.

Statement #1

**Silvia believes that her engagement ring is at the nail salon.**

**TRUE**

FALSE

Statement #2

**Silvia does NOT think that she left her engagement ring at the nail salon.**

TRUE

**FALSE**

Statement #3

**Silvia knows that if she were to go to the nail salon, she would find her engagement ring.**

**TRUE**

FALSE

Statement #4

**If Silvia learns that her engagement ring is NOT in the nail salon, she would NOT feel the following emotion:**

SURPRISED

ANGRY

**HAPPY**

SAD

AFRAID

DISGUSTED

Statement #5

**Silvia will go to the nail salon to retrieve her engagement ring.**

**TRUE**

FALSE

**Low-Uncertainty**

Introduction. Meet Sylvia. Sylvia is a 23-year-old administrative assistant at a popular car insurance company. Sylvia’s long-time boyfriend Sean recently proposed to her with a 2-carat princess-cut engagement ring.

Consistent 1. Last Thursday, Sylvia drove 3 and a half hours to visit her college roommate Bethany. Sylvia and Bethany got their nails done on Friday morning at a local nail salon in Bethany’s town. Sylvia requested a hand-exfoliation massage so she removed her engagement ring and placed it on the counter at the salon.

Consistent 2. Sylvia wasn’t used to taking off her engagement ring and found it difficult to remove it. When she did, she looked at her engagement ring on the counter admiringly.

Consistent 3. On her way home, Sylvia noticed how clean her hands look and immediately, it occurred to her that she’s missing her engagement ring! In a panic, Sylvia pulled over, called Bethany, and asked for the nail salon’s telephone number.

Consistent 4. When Bethany asked “are you sure you left it at the nail salon?” Sylvia replied with a quick “yes, I’m positive”.

Consistent 5. When she spoke with the receptionist at the nail salon, Sylvia told her the following: “Hello, my name is Sylvia Rosenberg. I was in for an exfoliating hand massage and manicure a few hours ago and I’m certain that I have left my engagement ring on your front desk”.

Consistent 6. After a few moments, Sylvia turned the key in her ignition, and drove towards the nail salon.

Statement #1

**Silvia believes that her engagement ring is at the nail salon.**

**TRUE**

FALSE

Statement #2

**Silvia does NOT think that she left her engagement ring at the nail salon.**

TRUE

**FALSE**

Statement #3

**Silvia knows that if she were to go to the nail salon, she would find her engagement ring.**

**TRUE**

FALSE

Statement #4

**If Silvia learns that her engagement ring is NOT in the nail salon, she would NOT feel the following emotion:**

SURPRISED

ANGRY

**HAPPY**

SAD

AFRAID

DISGUSTED

Statement #5

**Silvia will go to the nail salon to retrieve her engagement ring.**

**TRUE**

FALSE

**High-Uncertainty**

Introduction. Meet Sylvia. Sylvia is a 23-year-old administrative assistant at a popular car insurance company. Sylvia’s long-time boyfriend Sean recently proposed to her with a 2-carat princess-cut engagement ring.

Consistent 1. Last Thursday, Sylvia drove 3 and a half hours to visit her college roommate Bethany. Sylvia and Bethany got their nails done on Friday morning at a local nail salon in Bethany’s town. Sylvia requested a hand-exfoliation massage so she removed her engagement ring and placed it on the counter at the salon.

Consistent 2. Sylvia wasn’t used to taking off her engagement ring and found it difficult to remove it. When she did, she looked at her engagement ring on the counter admiringly.

Consistent 3. On her way home, Sylvia noticed how clean her hands look and immediately, it occurred to her that she’s missing her engagement ring! In a panic, Sylvia pulled over, called Bethany, and asked for the nail salon’s telephone number.

Inconsistent 1. While waiting for Bethany to give her the nail salon’s telephone number, Sylvia searched through her purse for her engagement ring.

Inconsistent 2. She then closed her eyes and traced back her steps to locate precisely the last place that she remembers leaving her engagement ring.

Inconsistent 3. A few moments later, Sylvia turned the key in her ignition and began driving.

Statement #1

**Silvia believes that her engagement ring is at the nail salon.**

**TRUE**

FALSE

Statement #2

**Silvia does NOT think that she left her engagement ring at the nail salon.**

TRUE

**FALSE**

Statement #3

**Silvia knows that if she were to go to the nail salon, she would find her engagement ring.**

**TRUE**

FALSE

Statement #4

**If Silvia learns that her engagement ring is NOT in the nail salon, she would NOT feel the following emotion:**

SURPRISED

ANGRY

**HAPPY**

SAD

AFRAID

DISGUSTED

Statement #5

**Silvia will go to the nail salon to retrieve her engagement ring.**

**TRUE**

FALSE

**Vignette #15**

**Intermediate-Uncertainty**

Introduction. Emma is a fourth-year Political Science major at Northwestern University. Every morning, Emma drinks a cup of coffee while reading the daily newspaper.

Filler 1. Emma’s younger sister Miranda is currently studying drama at Northwestern and so, Emma and Miranda live together in a downtown duplex.

Filler 2. As part of her coursework, Miranda must perform in various live plays throughout the academic year.

Consistent 1. The political climate in the United States of America is currently tense, as there is a well-publicized presidential election underway. The two main political parties are now lead by candidate O’Grady and candidate Leonard. Emma recently gave a passionate speech in one of her political science classes about candidate O’Grady’s potential as a great leader of their country.

Consistent 2. When researching candidate Leonard’s political views, Emma found herself shaking her head and rubbing her temples.

Filler 3. Every Saturday Emma accompanies Miranda to one of her drama recitals and applauds encouragingly after each scene.

Filler 4. Last Saturday, Miranda performed a scene from a literary classic—“Romeo and Juliet”.

Statement #1

**Emma does NOT believe that candidate O'Grady would make a good president.**

TRUE

**FALSE**

Statement #2

**Emma thinks that candidate O'Grady's opponent, candidate Leonard, will make a better president**

TRUE

**FALSE**

Statement #3

**Emma knows that if candidate O'Grady were to lose the election, it would be a loss for all the citizens of the United States of America.**

**TRUE**

FALSE

Statement #4

**If candidate O'Grady were to win the election, how would Emma feel?**

SURPRISED

ANGRY

**HAPPY**

SAD

AFRAID

DISGUSTED

Statement #5

**Emma will NOT vote for candidate O'Grady on voting day.**

TRUE

**FALSE**

**Low-Uncertainty**

Introduction. Emma is a fourth-year Political Science major at Northwestern University. Every morning, Emma drinks a cup of coffee while reading the daily newspaper.

Consistent 1. The political climate in the United States of America is currently tense, as there is a well-publicized presidential election underway. The two main political parties are now lead by candidate O’Grady and candidate Leonard. Emma recently gave a passionate speech in one of her political science classes about candidate O’Grady’s potential as a great leader of their country.

Consistent 2. When researching candidate Leonard’s political views, Emma found herself shaking her head and rubbing her temples.

Consistent 3. When Emma’s classmate Raphael remarked that he will most definitely vote for candidate O’Grady, Emma smiled and nodded her head.

Consistent 4. Emma watched candidate O’Grady’s speech on his potential as a leader on YouTube and hit the “like” button afterwards.

Consistent 5. Emma’s second-cousin Wesley plans on voting for candidate Leonard despite the fact that Emma has attempted to pursued him otherwise.

Consistent 6. When candidate O’Grady visited Evanston, Illinois, Emma attended his city hall speech and applauded as he declared his political plans for the United States of America.

Statement #1

**Emma does NOT believe that candidate O'Grady would make a good president.**

TRUE

**FALSE**

Statement #2

**Emma thinks that candidate O'Grady's opponent, candidate Leonard, will make a better president**

TRUE

**FALSE**

Statement #3

**Emma knows that if candidate O'Grady were to lose the election, it would be a loss for all the citizens of the United States of America.**

**TRUE**

FALSE

Statement #4

**If candidate O'Grady were to win the election, how would Emma feel?**

SURPRISED

ANGRY

**HAPPY**

SAD

AFRAID

DISGUSTED

Statement #5

**Emma will NOT vote for candidate O'Grady on voting day.**

TRUE

**FALSE**

**High-Uncertainty**

Introduction. Emma is a fourth-year Political Science major at Northwestern University. Every morning, Emma drinks a cup of coffee while reading the daily newspaper.

Inconsistent 1. The political climate in the United States of America is currently tense, as there is a well-publicized presidential election underway. The two main political parties are now lead by candidate O’Grady and candidate Leonard. After watching candidate O’Grady’s speech on his potential as a leader on YouTube, Emma hit the “dislike” button afterwards.

Inconsistent 2. Emma’s second-cousin Wesley plans on voting for candidate O’Grady despite the fact that Emma has attempted to pursued him otherwise.

Inconsistent 3. When candidate Leonard visited Evanston, Illinois, Emma attended his city hall speech and applauded as he declared his political plans for the United States of America.

Consistent 1. Emma recently gave a passionate speech in one of her political science classes about candidate O’Grady’s potential as a great leader of their country.

Consistent 2. When researching candidate Leonard’s political views, Emma found herself shaking her head and rubbing her temples.

Consistent 3. When Emma’s classmate Raphael remarked that he will most definitely vote for candidate O’Grady, Emma smiled and nodded her head.

Statement #1

**Emma does NOT believe that candidate O'Grady would make a good president.**

TRUE

**FALSE**

Statement #2

**Emma thinks that candidate O'Grady's opponent, candidate Leonard, will make a better president**

TRUE

**FALSE**

Statement #3

**Emma knows that if candidate O'Grady were to lose the election, it would be a loss for all the citizens of the United States of America.**

**TRUE**

FALSE

Statement #4

**If candidate O'Grady were to win the election, how would Emma feel?**

SURPRISED

ANGRY

**HAPPY**

SAD

AFRAID

DISGUSTED

Statement #5

**Emma will NOT vote for candidate O'Grady on voting day.**

TRUE

**FALSE**
