# Supplementary material for: Reasoning about mental states under uncertainty
Source: PLoS One. 2022 Nov 9;17(11):e0277356. doi: 10.1371/journal.pone.0277356 (PMC9645647; doi:10.1371/journal.pone.0277356)
Supplement: S2 Appendix — (DOCX) [file pone.0277356.s002.docx]

# Randomization Technique for Vignettes

With respect to content within the vignettes, we wanted to ensure that personal biases do not influence the topics of each story and so, all aspects of each story were generated randomly using the following four steps:

1. Each story consisted of a character whose gender was decided using a random number generator (i.e. 1 = “female” and 2 = “male). The name of the character was decided using the following random baby name generator online: <http://www.randomnames.com/>.
2. The element onto which the character would have a set of mental states about could be a person, place, thing, or phenomenon and this was decided using a random number generator.
3. If it was a person, then the name and gender were decided in the same way that those of the first character were generated. If it was a place, thing, or phenomenon, then the following random generators online were used: <https://www.randomlists.com/randomworld-cities>, <https://www.randomlists.com/things>, and <http://www.ratespeeches.com/t=Speech-Topics>, respectively.
4. Once the element onto which the agent has a mental state was decided, using a random number generator, whether or not the character agrees, likes, wants (i.e. approaches) or disagrees, dislikes, hates (i.e. avoids) the element was generated randomly.

*For example, a male (step 1) named Lewis (step 2) has a set of mental states (beliefs, thoughts, knowledge, emotions, and behavioral intentions) about a second character named Clara’s piano playing (step 3); in particular, Lewis likes (step 4) Clara’s piano playing.*

# Technique for Topic Matching

Despite the fact that vignette topics were generated using the randomization technique outlined in above—it is still possible that story topics for low-uncertainty vignettes (by chance) happen to be simpler than those in, say, high-uncertainty vignettes. We therefore decided to take an additional precaution to ensure that story topics are matched across conditions. Thus, for each story topic (i.e. the story about Lewis), we generated a low-uncertainty, intermediate-uncertainty, and high-uncertainty version. Participants each saw a total of fifteen vignettes, five of which were low-uncertainty story versions, five of which were intermediate-uncertainty story versions, and five of which were high-uncertainty story versions.

# Generation of Congruency Score

To obtain the continuous dependent variable (a congruency score) for each condition from participants, we obtained their responses to each of the five (believe, think, know, emotion, and intention) test questions presented after each vignette. For the low-uncertainty condition, for example, each participant read five low-uncertainty vignettes after which five questions were asked. Therefore, for this condition, participants each provided responses to a total of twenty-five questions—resulting in a calculated score out of twenty-five for the low-uncertainty condition. The same logic was applied to the intermediate and high-uncertainty conditions.
