# Supplementary material for: Reasoning about mental states under uncertainty
Source: PLoS One. 2022 Nov 9;17(11):e0277356. doi: 10.1371/journal.pone.0277356 (PMC9645647; doi:10.1371/journal.pone.0277356)
Supplement: S3 Appendix — (DOCX) [file pone.0277356.s003.docx]

**S3 Appendix. Reaction Time Data Analyses**

**Experiment 1**

A repeated-measures ANOVA revealed that there is a statistically significant effect of condition on RT (see Fig S1), *F*(2, 110) = 24.12, *p* < .001, ηp^2^ = 0.31, BF_10_ = 4.63e+6. Follow-up pairwise comparisons are provided in Table S1. RT in the intermediate-uncertainty condition (*M* = 5191.73; *SD* = 1744.01) was not significantly different than that in the low-uncertainty condition (*M* = 5043.65; *SD* = 1833.75), BF_01_ = 4.65. However, all other conditions significantly differed from one another, such that RT was greater in the high-uncertainty condition (*M* = 6129.64; *SD* = 2336.26) than either the intermediate- or low-uncertainty conditions.

**Fig S1. Reaction times as a function of uncertainty in Experiment 1. Error bars represent standard error.**

**Table S1.** Results of the Bonferroni-corrected Pairwise Comparisons between the Three Conditions of Experiment 1.

| Comparisons | Mean difference | 95% Confidence Interval for Difference [Lower, Upper] | p-value (Bonferroni-corrected) | BF_10,U_* |
| --- | --- | --- | --- | --- |
| Low vs. Intermediate Uncertainty | -148.08 | [-552.42, 256.27] | 1.00 | 0.215 |
| Intermediate vs. High Uncertainty | -937.91 | [-1360.94,  -514.88] | <.001 | 14448.53 |
| Low vs. High Uncertainty | -1085.99 | [-1514.66,  -657.31] | <.001 | 220004.81 |

*Note.* BF_10,U_* = Uncorrected Bayes Factor

**Experiment 2**

A repeated-measures ANOVA revealed that there is a statistically significant effect of condition on RT (see **Fig S2**), *F*(1.676, 77.09) = 5.82, *p* = .007, ηp^2^ = 0.11, BF_10_ = 7.93. Follow-up pairwise comparisons are provided in Table S2. RT in the Intermediate-quantity condition (*M* = 5342.91; *SD* = 1904.73) was not significantly different than that in the high-quantity condition (*M* = 5061.94; *SD* = 1618.18), BF_01_ =2.57. However, all other conditions significantly differed from one another, such that RT was greater in the low-quantity condition (*M* = 5725.52; *SD* = 2037.39) than either the intermediate or low-uncertainty conditions.

**Fig S2. Reaction times as a function of quantity in Experiment 2. Error bars represent standard error.**

**Table S2.** Results of the Bonferroni-corrected Pairwise Comparisons between the Three Conditions of Experiment 2.

| Comparisons | Mean difference | 95% Confidence Interval for Difference [Lower, Upper] | p-value (Bonferroni-corrected) | BF_10,U_* |
| --- | --- | --- | --- | --- |
| High-quantity vs. Intermediate-quantity | -280.97 | [-782.71, 220.77] | .512 | 0.39 |
| Intermediate-quantity vs. Low-quantity | -382.61 | [-743.63,  -21.59] | .034 | 3.42 |
| High-quantity vs. Low-quantity | -663.58 | [-1232.99,  -94.17] | .017 | 6.18 |

*Note.* BF_10,U_* = Uncorrected Bayes Factor

**Experiment 3**

A repeated-measures ANOVA revealed that there is a statistically significant effect of condition on RT (see **Fig S3**), *F*(2, 90) = 26.85, *p* < .001, ηp^2^ = 0.37, BF_10_ = 1.23e+7. Follow-up pairwise comparisons are provided in Table S3. RT in the intermediate-consistency condition (*M* =6137.27; *SD* = 2244.63) was not significantly different than that in the low-consistency condition (*M* = 6608.34; *SD* = 2330.11), BF_01_ = 0.85. However, all other conditions significantly differed from one another, such that RT was faster in the high-consistency condition (*M* = 5102.40; *SD* = 1625.65) than either the intermediate or low-uncertainty conditions.

**Fig S3. Reaction times as a function of consistency in Experiment 3. Error bars represent standard error.**

**Table S3.** Results of the Bonferroni-corrected Pairwise Comparisons between the Three Conditions of Experiment 3.

| Comparisons | Mean difference | 95% Confidence Interval for Difference [Lower, Upper] | p-value (Bonferroni-corrected) | BF_10,U_* |
| --- | --- | --- | --- | --- |
| High-consistency vs. Intermediate-consistency | -1034.87 | [-1582.54, -487.21] | <.001 | 840.76 |
| Intermediate-consistency vs. Low-consistency | -471.07 | [-1029.22,  87.08] | .124 | 1.18 |
| High-consistency vs. Low-consistency | -1505.94 | [-1962.62,  -1049.27] | <.001 | 5.90e+7 |
