# Supplementary material for: Reasoning about mental states under uncertainty
Source: PLoS One. 2022 Nov 9;17(11):e0277356. doi: 10.1371/journal.pone.0277356 (PMC9645647; doi:10.1371/journal.pone.0277356)
Supplement: Attachment — Submitted filename: Response to Reviewers 23Sep2022.docx [file pone.0277356.s005.docx]

**RESPONSE TO REVISIONS**

1. **Reviewer 2 Comment: The definition of “accurate” answers**

*"You have addressed to all of the reviewers' point. However, the response to Reviewer 2's first and "primary" concern is not sufficiently convincing. That point is absolutely crucial for the interpretation of the study results. A way to disentangle the issue about what the "correct" answer actually is, and how participants actually weigh the available pieces of information, is to quantify them."*

**Response 1.** We agree completely with this comment and have made an important revision to the terminology of the dependent variable in order to address this point more thoroughly. In terms of the definition of “accurate” answers, we had detailed in the manuscript that we predefined accurate responses as those corresponding to the answers when only consistent information was provided. Therefore, as Reviewer 2 pointed out, in the high-uncertainty condition (4 inconsistent statements and 2 consistent statements), participants’ reasonable answers should be the answers corresponding to inconsistent information. Considering our definition of accurate answers mentioned earlier, participants’ responses in the high-uncertainty condition does not mean that their responses were “wrong" or "incorrect.” It reflects the extent to which their responses correspond to the predetermined set of mental states of the character in the vignette. Reviewer 2’s point is highly pertinent and in line with our definition of the dependent variable. Although we added a considerable amount of detail and explanation of this outcome in our original set of revisions, upon further reflection and in light of this round of re-revision, we have changed the term "accuracy" to "congruency score" to more closely reflect that the score is indicative of how congruent the response is to our predetermined set of mental states for the character. This way, a low congruency score does not convey the notion that participants are scoring poorly on mental state reasoning, but that their score is simply less congruent with our preset mental states. We feel that this change in terminology conveys a more neutral definition of participant scores than the previous term "accuracy", which implied that participants who score lower are inaccurate or poor in mental stat reasoning abilities. We would once again like to thank the Editor and Reviewer 2 for initiating this important revision. We have added the following:

Congruent scores were specified as those corresponding to the answers when only consistent information was provided. For example, at the outset of the trial, the experimenters pre-set a high "congruency score" to reflect scores indicating that the character Lewis likes Clara's piano playing. Thus, lower congruency scores in the high uncertainty condition do not indicate poor ToM ability, but rather, that participants’ responses reflected the information provided, which is inconsistent with the pre-set “congruent” response.

***Location: Page 11, Line 226-231***

["Accuracy" term renamed to the more appropriate term, "Congruency Score"]

***Locations: Throughout Manuscript, Figure Y-Axis Labels, & S2 Appendix "Generation of Congruency Score" Section***

2. **Editor and Reviewer 2’s Comment: How participants actually weight the pieces of information**

*"For now, you only have an ordinal classification (low, intermediate, high) based on your own judgement alone. You might collect some additional rating data from naive participants judging not only if, but also how much each single piece of information is consistent and indicative of specific states of minds. In this way, you can quantify, for each single trial, how much and how consistent the available information is."*

**Response 2.** Another point that the Editor and Reviewer 2 raised is with regards to the stimuli used in the experiments; the suggestion was to collect more data to ensure whether (a) each statement is genuinely consistent (or inconsistent) with the preset mental states of the character and (b) participants weightings of each of the pieces of information in each vignette to determine how informationally rich each statement is. To help illustrate this excellent comment, we've pasted below a sample vignette used in the study for reference.

**Sample Vignette from Experiment 1**

**[Introduction:]** Lydia is a dental assistant. Lydia recently got engaged to Andrew, her high-school sweetheart.

**[Consistent 1.]** Most of the time, Lydia keeps the temperature in her and Andrew’s house relatively cool, at about 10 degrees Celsius (or 50 degrees Fahrenheit).

**[Consistent 2.]** In the dental office, Lydia’s colleagues constantly complain about how cold she sets the temperature.

**[Consistent 3.]** Even in the winter, Lydia often sleeps without a blanket.

**[Consistent 4.]** When discussing possible honeymoon locations, Lydia suggested that she and Andrew consider a winter resort.

**[Consistent 5.]** One day at work, Lydia came across an advertisement for Jamaica as a honeymoon destination, she looked up the average weather in Jamaica. After learning that the temperature in Jamaica during their honeymoon will reach as high as 33 degrees Celsius (91 degrees Fahrenheit), Lydia raised her eyebrows.

**[Consistent 6.]** With a large red marker, Lydia drew a large “X” on the ad for Jamaica.

For example, "Consistent Statement #4" can be more informationally rich than "Consistent Statement #2" in making the assertion that Lydia will *not* choose Jamaica for their honeymoon.

Regarding point (a), we completely agree that additional data collection (i.e., asking naïve participants to rate whether each statement is consistent or inconsistent) would be helpful as a stimulus manipulation check, but unfortunately, we do not currently have the resources to collect more data. The resources required for such a feat include the availability of independently initiating/continuing research ethics board approval as well as funds to conduct another series of 3 follow-up experiments. We have thought critically and reflected carefully to explore our available options to address this. Considering our limited resources and feasibility constraints, a reasonable solution is to include a detailed subsection in the limitations section of the discussion devoted to discussing this transparently. We have also included a corresponding section in the future directions section with the trial outlined in detail as an avenue for future research. Moreover, our stimuli are submitted with our manuscript, allowing future researchers the means to conduct these experiments. However, please note that (even without the further data collection for manipulation check) the current data still show that the amount and consistency of information *influence* participants’ uncertainty and decisions regarding another person’s mental states. The added sections are as follows, with bolded sections indicating subheadings in the manuscript:

**Limitations and Future Directions**

[...]

Importantly, the stimuli and sentences themselves are wanting of a manipulation check to ensure that they are, in fact, consistent (or inconsistent) and if so, how much each sentence contributes to participants' mental state inferences. A follow-up study that further investigates whether and how participants weigh the available pieces of information will be necessary to clearly address the limitation. For example, using a rating design in which asking participants to quantify how much each single piece of information is consistent will elaborate the current findings. In this way, one could quantify, for each single trial, how much and how consistent the available information is.

***Location: Page 33, Line 713-720***

Regarding point (b), we were impressed with your great suggestion to design the experiment to quantify how helpful each statement in the vignettes is in cuing participants to their mental state inference. Based on the current task design and dataset, we have minimized the possible effect of different richness levels of each statement on the manipulation through the randomization process. Moreover, with 45 separate vignettes each consisting of 6 sentences, the likelihood that a particular sentence is driving any effects is low. Having said that, we agree that your suggestion of more data collection with a rating design can clearly ensure the consistency levels of each story, which is why we added these details to the Limitations and Future Directions sections (as noted above). We feel that these additions provide the reader with a deeper appreciation for our task, while being aware of its limitations and future experiments that can help resolve and improve its utility. This comment provided us with an opportunity to reflect on the virtues and merits of our stimuli, which we have now added as follows:

Despite the limitations outlined in the stimuli used within the three experiments of the current study, there are several features of the stimuli that are noteworthy. Contrary to other existing ToM tasks in which one definite correct answer exists (e.g., Sally-Anne task in which participants are asked to infer another agent’s mental state on an object’s location; location A or B?), the type of ToM tasks used in the current study (i.e., participants are asked to infer the mental state of others on their attitudes, beliefs, and emotions towards something or someone, which can be mixed or multiple), there are a plethora of opportunities to infer mental states with multiple questions targeting various mental state processes. This process provided us with a richer response for our primary outcome (i.e., rather than "location A/B" in previous ToM tasks) and is more reflective of mental state reasoning in every day, real life processes. That is, we typically do not simply ask ourselves a binary question pertaining to another individual's mental states. But rather, we integrate the complexities of people's mental states with a series of inferences on what the other person thinks, how they feel, what they believe to be true, and how they will likely behave in the future. We are also often faced with competing and complex information that may alter our mental state inferences, features which our task and manipulations capture. Furthermore, with our task, there can always be individual differences in which statement can be perceived as more rich to infer others’ mental states. To address this in the current set of studies and rule out any individual difference factors, we randomized many aspects of the stimuli including which sentences were chosen to appear in the vignettes of the other conditions. Therefore, it is unlikely that one informationally rich statement drove any effects observed, as the likelihood of each sentence being chosen in other conditions was random, as was the likelihood of a participant being exposed to each experimental condition.

***Location: Pages 34-35, Lines 742-763.***

3. **Data Analysis Suggestion**

*"A related point concerns the data analysis. Once you have quantified the amount and consistency of information available for each trial and you can use this information as predictors, you should analyze the data using statistical methods more appropriate than Anovas with normality assumptions. Since the actual responses are binomial (correct/incorrect) and they are repeated by participants and by trial/scenario, using mixed-effects logistic regressions is recommended. (This is recommended even if you do not quantify amount and consistency of information and you keep the independent variable as it is now.)"*

**Response 3.** And finally, conducting a binomial mixed-effects logistic regression to analyze the data was suggested. We agree that this suggestion would be most appropriate with binomial data, but our current accuracy measure is continuous. We have added the following to clarify that our dependent variable is operationalized as a continuous variable in the manuscript (bolded statement indicates new additions for illustrative purposes in this letter).

To address the hypothesis of a direct effect of the uncertainty manipulation on responses to questions (**as a continuous dependent variable**) pertaining to the agent's mental states, a repeated-measures ANOVA is required (with follow-up pairwise tests if applicable).

***Location: Page 7, Line 158-161.***
